# A review of formulations and preclinical studies of inhaled rifampicin for its clinical translation

**DOI:** 10.1007/s13346-022-01238-y

**Published:** 2022-09-21

**Authors:** Prakash Khadka, Jack Dummer, Philip C. Hill, Rajesh Katare, Shyamal C. Das

**Affiliations:** 1 School of Pharmacy, University of Otago, Dunedin, 9054 New Zealand; 2 Department of Medicine, Dunedin School of Medicine, University of Otago, Dunedin, 9054 New Zealand; 3 Centre for International Health, Department of Preventive and Social Medicine, Dunedin School of Medicine, University of Otago, Dunedin, 9054 New Zealand; 4 Department of Physiology, HeartOtago, School of Biomedical Sciences, University of Otago, Dunedin, 9054 New Zealand

**Keywords:** Tuberculosis, Inhalation, Rifampicin, Preclinical development, Animal models

## Abstract

Inhaled drug delivery is a promising approach to achieving high lung drug concentrations to facilitate efficient treatment of tuberculosis (TB) and to reduce the overall duration of treatment. Rifampicin is a good candidate for delivery via the pulmonary route. There have been no clinical studies yet at relevant inhaled doses despite the numerous studies investigating its formulation and preclinical properties for pulmonary delivery. This review discusses the clinical implications of pulmonary drug delivery in TB treatment, the drug delivery systems reported for pulmonary delivery of rifampicin, animal models, and the animal studies on inhaled rifampicin formulations, and the research gaps hindering the transition from preclinical development to clinical investigation. A review of reports in the literature suggested there have been minimal attempts to test inhaled formulations of rifampicin in laboratory animals at relevant high doses and there is a lack of appropriate studies in animal models. Published studies have reported testing only low doses (≤ 20 mg/kg) of rifampicin, and none of the studies has investigated the safety of inhaled rifampicin after repeated administration. Preclinical evaluations of inhaled anti-TB drugs, such as rifampicin, should include high-dose formulations in preclinical models, determined based on allometric conversions, for relevant high-dose anti-TB therapy in humans.

**Graphical abstract:**

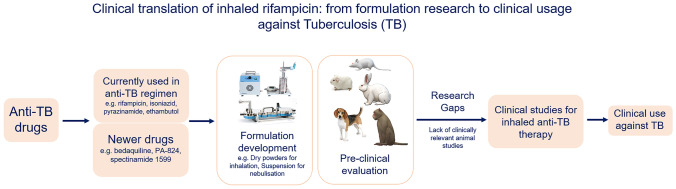

## Introduction

### Current status of TB and approach to TB treatment

TB is caused by *Mycobacterium tuberculosis* (Mtb) and mainly affects the lungs, but can also affect extra-pulmonary sites. Mtb is transmitted when aerosols from an infected person are inhaled by another person. The World Health Organization (WHO), in its 2021 TB report, stated that TB was the top cause of death worldwide from a single infectious agent in 2019 [1]. There were 5.8 million new TB cases diagnosed and reported in 2020, among which approximately 1.3 million cases were fatal [1]. A decline in the number of new TB cases and an increase in the number of TB deaths, compared to those in 2019, was observed in 2020 due to the COVID-19 pandemic that resulted in reduced access to TB diagnosis and treatment [1]. The majority of TB illnesses occur in low-resource countries, and over 85% of new TB cases occur in the 30 countries with the highest TB burden [1].

The recommended treatment for drug-susceptible TB is a 6-month rifampicin-based regimen of orally administered drugs, which includes the administration of isoniazid (H), rifampicin (R), pyrazinamide (Z), and ethambutol (E) (HRZE) for 2 months (daily or thrice weekly), followed by 4 months of HR [2]. Treatment regimens have become more complicated with the rise in complications of TB, such as multi-drug resistant TB (MDR-TB), co-infection with HIV, comorbidities such as diabetes, or TB retreatment after relapse. Approaches to treating drug-resistant TB may involve the inclusion of an injectable antibiotic, such as streptomycin, kanamycin, and amikacin, or lengthening treatment duration up to 20 months with drugs to which the organism is susceptible [3]. The real-world success rates of TB treatment regimens are lower than expected due to various factors such as low patient adherence to the lengthy treatment regimen, the existence of Mtb deep inside human TB lesions, and the latency of Mtb facilitating its survival within the host by up- and down-regulation of specific genes [4].

Mtb usually resides within complex granulomatous lesions, which allow the pathogen to avoid the bactericidal effects of antibiotics by limiting their transportation from blood to the bacterial target site and contribute to treatment failure, relapse of disease, and the emergence of drug-resistant strains of the bacteria [4, 5]. This presents a hurdle for efficient drug delivery to the complex lesions of pulmonary TB via the oral route. This also requires the administration of high drug doses since only a small fraction of the orally administered dose reaches the target sites in the lungs [6]. Moreover, there is a risk of adverse effects from the chronic administration of drugs.

Among the many reasons for the spread of MDR-TB is chaotic treatment exacerbated by poor health care systems, the amplifier effect of short-course chemotherapy, community transmission, and nosocomial transmission in places with high TB burdens [7]. Moreover, exposure of the mycobacteria to sub-therapeutic levels of anti-TB drugs during treatment is a driver for the emergence of drug-resistant microbial strains [8]. One effective approach to increase the bactericidal effect of the antibiotics is to achieve a high local concentration of the drug by localized drug delivery, such as pulmonary administration.

As outlined above, TB treatment remains a challenge despite the availability of potent drugs, and this has driven a search for clinically efficacious treatment approaches that can shorten treatment duration. There has been a huge investment in the development of newer anti-TB drugs, with several drugs already in the ‘drug development pipeline’ and at various stages of clinical trials [9]. These drug candidates are promising and have novel targets to achieve the efficient bactericidal activity, but their journey from the initial drug development phase and clinical trials to clinical practice is still a few years away [9, 10]. TB treatment approaches should also focus on better delivery options for drugs that are currently available and are effective against Mtb.

### Pulmonary drug delivery approach for TB treatment: evidence from preclinical and clinical studies

Pulmonary delivery of anti-TB drugs is expected to deliver high drug concentrations at the desired site, i.e., to the granulomatous TB lesions as well as the systemic circulation [11, 12]. The pulmonary route can be exploited for its advantages, such as the large surface area of the lungs, rich vascularization, and thin barrier to systemic circulation, which allows noninvasive administration of drugs for local and systemic effects while avoiding first-pass metabolism and achieving rapid onset at the same time, during TB treatment [13]. Both local and systemic effects of the drugs are desirable in inhaled TB therapy because achieving adequate drug levels in the blood, together with that in the lungs, is expected to achieve better therapeutic results in both pulmonary and extra-pulmonary TB.

Pulmonary delivery of antibiotic therapy has already been used effectively in other clinical settings to treat infection and combat drug resistance. Clinical studies in respiratory intensive care unit patients have shown that aerosolized antibiotics can eradicate pathogens causing respiratory infection and reduce the incidence of newly resistant organisms [14, 15]. Mtb is a major pulmonary pathogen with a high incidence of multidrug resistance [16, 17], and inhaled therapy has the potential to reduce resistance by ensuring maximum concentration of the drug at the target site for efficient bacterial killing [18]. The pulmonary route increases drug delivery to TB lesions in the lungs and drug retention. The pulmonary route can also be used for systemic drug delivery to treat extra-pulmonary TB. Ideally, aerosol particles of the inhalable size range (1–5 µm in diameter) are desired for deep lung delivery, even though smaller particles such as the nanoparticles serve the purpose better for intracellular targeting and uptake by the macrophages. Therefore, approaches such as nano-in-micro particle systems have been used to achieve both good aerosolization as well as improved cellular uptake after deposition in the lungs [19].

Pulmonary delivery of drugs to the lungs can be achieved in several ways: by aerosolization of drug powder particles using dry powder inhalers (DPI), by nebulization of a drug solution or suspension using nebulizers, by liquid aerosolization using metered dose inhalers (MDIs), and by direct drug administration to the upper respiratory tract using insufflators [6]. Due to the requirement of high drug doses in TB treatment and the application of MDIs being limited to aerosolization of low drug doses, MDIs have not been researched for this purpose [20]. Nebulizers and DPIs of drugs for several other respiratory diseases, such as chronic obstructive pulmonary disease (COPD) and asthma, are well-established clinically. DPIs are preferred for their convenience in usage during inhalation delivery; their advantages include propellant-free formulations, minimized formulation-related problems, and a lesser need for patient coordination [21, 22]. Compared to formulations for nebulization, DPI formulations offer better stability of drug products due to their solid nature, are convenient, and allow quick administration, while nebulization requires a significant duration of time for the administration.

Several current and new anti-TB drugs have been studied for their inhalable formulation development, in vitro and in vivo evaluation, and only one of them has been studied in humans but at a subtherapeutic dose [23, 24]. Preclinical studies reporting on inhaled drugs for TB treatment suggest that inhaled delivery can ensure higher drug concentrations in the lung and achieve better therapeutic effects against TB with lower doses and toxicity, compared to oral and injectable administration. An investigation of inhaled rifampicin formulations in rats [25] suggested an increased drug concentration in the alveolar macrophages from intra-tracheal administration compared to oral administration. Similar results were observed in the case of a dry powder formulation of isoniazid [26]. In an infectious disease model [27], respirable microspheres containing rifampicin showed promise in reducing the burden of bacteria in the lungs. Another antitubercular drug, pyrazinamide, was found to achieve an absolute bioavailability of 66% when drug-loaded large porous particles were administered to guinea pigs by intra-tracheal insufflation [28]. In vitro efficacy and in vivo safety of repeated intra-tracheal administration of the same drug have also been reported [29]. Capreomycin is another promising drug candidate for inhaled delivery in the treatment of pulmonary tuberculosis. Inhaled capreomycin has been reported to have low toxicity from in vivo acute toxicity studies [30]. Another example of a promising inhaled anti-tubercular drug is ofloxacin, which has also demonstrated improved treatment efficacy against tuberculosis following pulmonary administration compared to other routes of administration, such as the intravenous and the oral route [31]. Newer drugs such as PA-824, Spectinamide-1599, All-trans-retinoic acid, and para-aminosalicylic acid also have shown promise as inhaled therapies in animal studies. Large porous particles of PA-824 were reported to achieve high drug concentrations in the lungs after pulmonary administration compared to oral administration to guinea pigs [32]. Similarly, guinea pigs with experimental TB showed lower inflammation, bacterial burden, and tissue damage when treated with a dry powder inhaled formulation of PA-824 compared to nontreated or placebo groups [33]. A study conducted in mice reported the reproducibility of dry powder administration of a spray-dried formulation of Spectinamide-1599, also demonstrating dose-dependent exposure of this promising anti-TB drug [34]. The efficacy of an inhaled formulation of all trans-retinoic-acid (ATRA) was reported in mice in which the inhaled treatment significantly reduced the bacterial burden and pulmonary pathology following just three doses [35]. In another study, direct delivery of para-aminosalicylic acid to the rat lungs was reported to achieve rapid and higher local concentration and similar systemic concentration of the drug, compared to oral delivery [36].

Only a few clinical studies on inhaled anti-TB drugs have been reported, and they include studies on rifampicin and capreomycin dry powder inhalers. A clinical study of a low dose (2 mg) of rifampicin delivered as a dry powder inhaled formulation [24] suggested that it did not provoke inflammatory cytokines and was safe to healthy subjects. In another study, the safety and pharmacokinetics of capreomycin delivered via dry powder inhaler was tested in humans up to 300 mg nominal doses [37]. The study reported safety to the lungs, rapid drug absorption after inhalation, and serum drug concentrations higher than the MIC of the drug for Mtb.

In the following sections, the formulations and animal studies reported in the literature for inhaled rifampicin are reviewed, and the considerations for preclinical studies of inhaled anti-TB drugs are discussed with a focus on inhaled rifampicin.

## Rifampicin: a long-established oral anti-TB drug with new potential via the inhaled route

Rifampicin (also known as rifampin) is a semisynthetic derivative of rifamycin B that exhibits its bactericidal effect on mycobacteria by inhibiting their DNA-dependent RNA synthesis [38]. It was discovered in 1965 as a promising rifamycin derivative for oral use and was approved for oral delivery against pulmonary TB by the USFDA in 1971. Since then, rifampicin has been central to TB treatment. Together with isoniazid and pyrazinamide, it is used as the first-line agent against both pulmonary and extra-pulmonary TB at a dose of 10 mg/kg/day [39]. This dose was chosen to avoid any dose-dependent toxicity and because of its expense when it initially entered clinical use [40]; however, it is now evident that higher doses of oral rifampicin are well tolerated by humans and are more effective than the conventional 10 mg/kg/day (maximum of 600 mg/day) doses in achieving higher drug concentration in the blood [41, 42].

Rifampicin is the most promising anti-TB drug for development as inhaled therapy due to its high efficacy in sterilizing TB lesions and preventing relapse, a relatively long history of use in humans, and well-documented pharmacokinetic and pharmacodynamic properties from the oral route. Rifampicin is also considered the best candidate for improving therapeutic outcomes in TB treatment if used at a higher oral dose than in current regimens. A dose-ranging clinical trial has already demonstrated that rifampicin doses up to 35 mg/kg from the oral route are safe as well as efficient in achieving high systemic drug concentration (AUC_0-24_ up to 235 h·mg/L and *C*_max_ up to 35.2 mg/L) and reducing time to sputum culture conversion with higher doses [42]. Several other clinical trials have also demonstrated the safety and/or efficacy of high-dose oral rifampicin [41, 43–45]. Therefore, rifampicin is likely to remain the backbone of anti-TB regimens for the years to come, and despite calls for higher oral doses, they have not yet been implemented for pulmonary TB in clinical practice [46].

High oral dose of rifampicin for TB treatment may be limited by the increased risk of systemic toxicities and there are challenges such as its unknown pharmacodynamics alone and in combination with other medications. Due to this reason, research has focused on other ways of delivering rifampicin more efficiently [46]. Delivering rifampicin by inhalation is a potential alternative to achieve high drug concentration in the lungs as well as systemic circulation with a smaller dose. After pulmonary delivery, high blood concentration can be achieved at a smaller dose than the oral dose due to rapid drug absorption through the large surface area and minimal drug metabolism in the lung [47, 48], which may reduce the likelihood of dose-dependent systemic toxicity of rifampicin. Inhaled rifampicin has a huge potential for clinical translation and could be used as an adjunct to current oral anti-TB regimens for improved therapeutic outcomes in TB patients.

### Inhaled rifampicin: formulation types and preparation techniques

The dry powder formulations of anti-TB drugs for inhaled therapy against TB have been extensively reviewed previously [20, 49, 50]. Similarly, Mehanna et al. and Chae et al. have reviewed inhalable nanoparticulate systems for anti-TB drugs [51, 52]. In the present review, we have focused on the different types of inhalable formulations of rifampicin. A wide range of anti-TB drugs has been investigated for their potential as inhaled formulations suitable for lung delivery, suggesting a huge interest among researchers for inhaled anti-TB therapy (Table 1). Among these, rifampicin is the most commonly studied, either alone or in combination with other drugs, and dry powder formulations for inhalation have been studied more frequently than nebulized formulations. Spray drying is the most common technique utilized to obtain the final inhalable formulation of dry powder rifampicin with good aerodynamic properties (Table 1), although other techniques include jet milling [53] and micronization [54].

**Table 1:** Inhalable formulations reported for anti-TB drugs and the type of study on such formulations

**Anti-TB drugs and their formulations**	**Type of study**	**Reference**
Spray dried particles of rifampicin	In vitro evaluation	[60, 112–114]
	In vitro and in vivo evaluation	[25]
	In vitro evaluation and screening in the disease model	[27, 115]
Spray dried particles of isoniazid	In vitro and in vivo evaluation	[26]
	In vitro evaluation	[116]
Spray dried particles of pyrazinamide	In vitro evaluation	[117]
	In vitro and in vivo evaluation	[28]
Spray dried particles of rifapentine	In vitro evaluation	[118–120]
	In vitro and in vivo evaluation	[29, 121]
Spray dried particles of ethambutol	In vitro evaluation	[122, 123]
Spray dried particles of capreomycin	In vitro evaluation	[124]
	Phase I clinical study	[37]
	In vitro and in vivo evaluation	[124, 125]
Spray dried particles of clofazimine	In vitro and in vivo evaluation	[126]
Spray dried particles of ofloxacin	In vitro characterization	[127]
	In vitro and in vivo evaluation	[31]
Spray dried particles of PA-824	In vitro evaluation and in vivo efficacy study	[33]
	In vitro and in vivo evaluation	[32]
Spray dried particles of kanamycin	In vitro evaluation	[128]
Spray dried particles of SHetA2	In vitro evaluation	[129]
Spray dried particles of Spectinamide-1599	In vitro evaluation and in vivo pharmacokinetics	[34]
Spray dried particles of All-trans-retinoic acid	In vitro and in vivo evaluation	[35]
Spray dried particles of para-aminosalicylic acid	In vitro and in vivo evaluation	[36]
Spray dried particles of rifampicin and isoniazid	In vitro and ex vivo studies	[130]
	In vitro evaluation	[131, 132]
	In vitro and in vivo evaluation	[71, 107]
Spray dried particles of rifampicin and rifabutin	In vitro and in vivo evaluation	[70]
Spray dried particles of rifampicin, pyrazinamide, and isoniazid	In vitro evaluation	[133]
Spray dried particles of isoniazid and rifabutin	In vitro and in vivo evaluation	[106]
	In vitro evaluation	[134, 135]
Spray dried particles of isoniazid and ciprofloxacin	In vitro evaluation	[110]
Spray dried particles of pyrazinamide and moxifloxacin	In vitro evaluation	[136]
Spray dried particles of bedaquiline, moxifloxacin, and pyrazinamide	In vitro evaluation	[137]
Spray dried particles of ethionamide and moxifloxacin	In vitro evaluation	[138]
Spray dried particles of D-cycloserine and ethionamide	In vitro evaluation	[139]
Spray dried particles of verapamil and rifapentine	In vitro evaluation	[140]
Spray dried particles of thioridazine and rifapentine	In vitro evaluation	[141]
Spray dried particles of pyrazinoic acid and pyrazinoic acid ester	In vitro evaluation and evaluation of an animal exposure chamber	[142]
Spray dried particles of rapamycin alone or in combination with isoniazid and rifabutin	In vitro evaluation and in vivo efficacy study	[143]
Spray dried particles of tobramycin, amikacin, and kanamycin	Inhaler design study	[144]
Spray dried particles of rifapentine, moxifloxacin, and pyrazinamide	In vitro evaluation	[145]
Spray dried particles of rifampicin, isoniazid, pyrazinamide, and levofloxacin	In vitro aerosolization study, safety in humans	[24]
Freeze dried particles of rifampicin	In vitro evaluation	[146, 147]
	In vitro and in vivo evaluation	[148]
	In vitro evaluation and ex vivo alveolar macrophage targeting	[149]
Freeze dried nanopowder of isoniazid	In vitro evaluation	[150]
Freeze dried particles of rifampicin, isoniazid, and pyrazinamide	In vitro and in vivo evaluation	[109]
Freeze dried particles of rifampicin, capreomycin, or para-aminosalicylic acid	In vitro evaluation	[151]
Spray dried nanoparticles of ethambutol	In vitro evaluation	[152]
Spray dried nanoparticles of rifampicin and isoniazid	In vitro and in vivo evaluation	[108]
Spray freeze dried particles of magainin-I analog peptide	In vitro evaluation	[19]
Micronized rifampicin and lactose blend	In vitro and in vivo evaluation	[54]
Jet milled particles of clofazimine	In vitro evaluation	[153]
Jet milled and spray dried particles of isoniazid	In vitro evaluation	[154, 155]
Freeze dried, vacuum dried and spray dried particles of isoxyl	In vitro evaluation	[156]
Vacuum dried and jet milled particles of rifampicin	In vitro evaluation	[53]
Vacuum dried nanoparticles of rifampicin, isoniazid, and pyrazinamide	In vitro and in vivo evaluation	[72]
Pressurized packed system of liposomal formulation of rifampicin	In vitro and in vivo evaluation	[63]
Spray dried nanocomposite of rifampicin	In vitro evaluation	[60]
Micellar systems of rifampicin	In vitro and in vivo evaluation	[55, 56]
Rifampicin soft pellets	In vitro characterization	[157]
Rifampicin loaded bovine serum albumin nanoparticles	In vitro evaluation	[59]
Rifampicin loaded metal–organic frameworks	In vitro evaluation	[158]
Isoniazid loaded metal–organic frameworks	In vitro evaluation	[159]
Drug-loaded nanoparticles of ethionamide	In vitro evaluation and in vivo efficacy study	[160]
Solid lipid nanoparticle dispersions of rifabutin	In vitro evaluation	[161]
Liposomal suspension of isoniazid	In vitro evaluation	[162]
Liposomal solution of rifampicin, isoniazid, and ethambutol	In vitro evaluation	[163]
Inhaled particles of rifampicin and isoniazid	In silico design	[164]

### Formulations for inhaled rifampicin

Various drug delivery systems are reported in the literature for inhaled delivery of rifampicin, such as microparticle systems, liposomes, solid lipid nanoparticles (SLN), polymeric nanoparticles, porous particles, nanoaggregates, and nanocomposites (Table 2). Recently, micellar systems, such as polymeric micelles and nanomicelles, also have gained attention as a promising formulation approach for inhaled delivery of rifampicin [55, 56]. Such formulations have shown good retention after intra-tracheal administration in animal models (Table 2). Although promising in enhancing drug bioavailability and localized retention, these carrier-based formulation systems need further investigation of their systemic absorption, degradation, and clearance from the body [57].

**Table 2:** Drug delivery systems for inhaled delivery of rifampicin: formulation features and in vitro and in vivo properties

**Drug delivery system**	**Formulation**	**Preparation technique**	**Aerosol properties**	**In vivo evaluation**	**Formulation features**	**Reference**
Micro-particle system	Dry powder for inhalation (DPI)	Spray drying and crystallization to obtain amorphous and crystalline rifampicin particles, respectively	Rifampicin powder particles with fine particle fraction (FPF) > 58% and mean particle size < 3.8 μm	N/A	Amorphous and crystalline dihydrate powder formulations of rifampicin with good aerosolization stability	[165]
	DPI	Micronized rifampicin mixed with coarse and fine lactose	Maximum fine particle fraction (FPF) of 28.9% and mass median aerodynamic diameter (MMAD) in the range of 4.3–5.8 μm	Toxicity and pulmonary pharmacokinetics	Negligible toxicity and higher lung levels compared to marketed formulation	[54]
	DPI	Chitosan microparticles prepared by ionic gelation technique followed by spray drying	FPF of 21.5% and MMAD of about 5 μm	Toxicity in rats	Sustained drug release up to 12 h and no local adverse effects	[70]
	DPI	Lipospheres prepared by spray drying rifampicin with *β*-cyclodextrin and vitamin C	FPF between 69.0–83.7% and MMAD of 1.8–4.0 μm	N/A	Lipospheres exhibited good aerodynamic properties and enhanced/equivalent antibacterial efficacy in vitro	[166]
	DPI	Lipospheres prepared by spray drying rifampicin with phospholipid	FPF 77.7% and MMAD 2.7 μm	Pulmokinetic and biodistribution studies in rats	Improved bioavailability and residence time of rifampicin in rat lungs	[167]
	DPI	Rifampicin microparticles prepared by spray drying	Aerosolization study not performed	Hepatotoxicity and phagocytosis activity in rats after intra-tracheal and oral administration	Intra-tracheal instillation of 5 mg/kg rifampicin led to high drug concentration in alveolar macrophages. No hepatotoxicity post intra-tracheal administration in rats	[25]
	DPI	Rifampicin dihydrate microcrystals prepared by polymorphic transformation of rifampicin were further coated with poly (DL-lactide-co-glycolide) or poly (DL-lactide) by spray drying	FPF of coated formulations were between 23.9 and 44.5%. MMAD between 3.5 and 4.5 μm	N/A	Carrier free formulation with sustained release property	[113]
	DPI	Rifampicin-loaded PLGA microspheres prepared by emulsion-solvent evaporation method followed by freeze-drying	MMAD of 4.5 μm and FPF of 52%	N/A	sevenfold higher uptake by alveolar macrophage compared to the free drug	[149]
	DPI as well as powder for resuspension prior to nebulization	PLGA-rifampicin microspheres prepared by solvent evaporation method	Aerosolization study not performed	Insufflation or nebulization of microspheres to guinea pigs with experimental TB followed by quantitative bacteriology and histopathological analyses	Reduction in viable bacteria, inflammation and lung damage in animals treated with RIF microspheres	[27]
Liposomes	Pressurized pack liposomal suspension	Rifampicin loaded liposomes prepared by lipid film hydration method were prepared into aerosolized packs	101–113 mg of liposomal suspension (45–55 μg rifampicin) was released per actuation	Alveolar macrophage deposition and tissue distribution in rats	Liposomal aerosols achieved high drug concentration in the lung with a high population of alveolar macrophages	[63]
	Suspension for nebulization	Liposomes prepared by thin film hydration method	FPF between 7 and 17% and nebulized fraction between 25 and 55% for all formulations	N/A	Liposomes prepared with high phosphatidylcholine concentration and cholesterol were the best candidates for inhaled rifampicin delivery	[61]
	Suspension for nebulization	Rifampicin loaded liposomes prepared by film hydration and freeze drying	Nebulization efficiency of all formulations was higher than 50% except for that prepared with soy lecithin	Determination of lung rifampicin levels in rats after nebulization	Liposomes were active against bacterial complex in infected macrophages in vitro and were able to reach lower airways in rats upon nebulization	[62]
	DPI	Liposomal powder prepared by chloroform‐film method followed by freeze drying	FPF 27.8–66.8% and MMAD 3.4–6.7 μm	N/A	Cholesterol improved stability of liposomal formulation and mannitol produced crystalline powder with good aerosol properties	[168]
Solid lipid nanoparticles (SLN)	DPI	SLN produced by melt emulsifying technique followed by freeze drying	Respirable fraction of 2.0–2.8% for functionalized and 17.5–25. 3% for non-functionalized SLN	N/A	Physical properties suitable for alveolar macrophage passive targeting	[146]
	DPI	SLN produced by melt emulsifying technique followed by freeze drying	FPF between 11.8 and 38.0% and aerodynamic diameter in the range of 210–676 nm	N/A	SLN were inhalable and showed active targeting to alveolar macrophages	[169]
Polymeric nanoparticles	Lyophilized powder	Cationic inhalable PLGA microparticles prepared by oil in water single emulsion method by freeze-drying	Aerosolization study not performed	Intratracheal instillation in mice followed by evaluation of macrophage targeting and in vivo imaging	Cationic microparticles led to improved particle uptake by macrophages and high intracellular accumulation of the drug both in vitro and in vivo	[170]
	Lyophilized powder	Chitosan-coated Alginate-Tween 80 nanoparticles prepared by ionic gelation followed by freeze drying	Aerosolization study not performed	N/A	Nanoparticles of rifampicin were less cytotoxic than free rifampicin. The nanoparticles showed significantly improved activity against nine clinical strains of Mtb	[147]
	DPI	Chitosan nanoparticles prepared by ionic gelation probe sonication method followed by freeze drying	FPF of 33.3% and MMAD of 3.3 μm	Pulmonary pharmacokinetics	Sustained release of drug up to 24 h, marked increase in half-life, maximum concentration and bioavailability compared to conventional formulations	[148]
	DPI	Amphiphilic lipopolymer system for rifampicin prepared by covalent conjugation and spray drying	FPF of 67.9% and MMAD of 2.3–2.4 μm	N/A	Biocompatible formulation, initial burst release followed by a controlled release for 24 h	[112]
	DPI	Rifampicin/ PLGA nanoparticles containing mannitol microspheres prepared by spray drying	Drug deposition in stages 2–7 on a cascade impactor was 35%	Uptake of rifampicin by alveolar macrophages in rat lungs	Enhanced uptake by alveolar macrophages in vivo	[114]
	Powder for resuspension prior to nebulization	PLGA nanoparticles prepared by multiple emulsion technique and vacuum drying	96% of the aerosolized particles were respirable (≤ 6 μm) with MMAD of 1.9 μm	Pharmacokinetics and chemotherapeutic potential evaluated in guinea pigs with experimental TB	6.5-fold increase in the absolute bioavailability of rifampicin from the nebulized formulation as well as an improved therapeutic effect in vivo compared to oral route	[72]
Porous particles	DPI	Rifampicin porous particles prepared by spray drying with leucine	FPF 52.9% and MMAD 4.8 μm	Pharmacokinetic study in guinea pigs	Half of the oral dose delivered via the inhaled route led to systemic concentrations similar to that after oral administration	[48]
	DPI	Rifampicin loaded PLGA nanoparticles were prepared by solvent evaporation method. The nanoparticles were then added to a solution of leucine and the suspension was spray dried to obtain porous particles containing nanoparticles	FPF 35.5 and 44.7% for particles containing 40 and 80% nanoparticles by weight, respectively. MMAD was 4.2 μm for both	Pharmacokinetic study in guinea pigs	Inhaled porous nanoparticle aggregated particles delivered rifampicin systemically and extended rifampicin levels in the lungs for up to 8 h	[171]
Nanoaggregates and nanocomposites	DPI	Spray drying of rifampicin nanosuspension	Respirable fraction up to 49.91%. Aerodynamic diameters between 1.46 and 2.99 μm	N/A	Significantly higher IC_50_ of rifampicin nanocomposite than the free drug	[60]
	DPI	Guar gum nanoporous aggregates prepared by precipitation technique followed by spray drying	Aerosolization study not performed	Organ distribution and pharmacokinetic studies in rats	Sustained release of drugs up to 48 h and predominant deposition of drugs within the lungs	[108]
Micellar systems	Suspension for nebulization	Preparation of rifampicin loaded polymeric micelles by solvent-diffusion technique	Fine particle fraction 57% and fine particle dose 6.2 mg upon in vitro nebulization study. MMAD was 3.86 μm	In vivo biodistribution assay after intra-tracheal administration in rats	High retention (84%) of the micelles in the body with 76% of that fraction accumulated in the lungs	[55]
	Suspension for nebulization	Nanomicelle formulation of rifampicin and curcumin for inhalation prepared using gelatin and mannose	Fine particle fraction 75%, fine particle dose 15 mg and aerodynamic diameter 1.26 μm for rifampicin only micelles upon in vitro nebulization study	In vivo micellar lung accumulation assay	After intra-tracheal administration, 45% dose reached the lungs in 1 h. After 24 h, a fraction of the intra-tracheal dose was found to be accumulated in the lungs	[56]

Rifampicin is also a good drug candidate for inhaled delivery after loading into micro and nano-carrier-based systems. A variety of inhalable carrier systems for rifampicin have been reported in the literature, ranging from polymers, lipids, non-polymeric carbohydrates, and mesoporous silica [58]. A nanoparticle system for rifampicin was reported by Joshi and Prabhakar in which bovine serum albumin was used to obtain nanoparticles for rifampicin loading [59]. In another study, a spray-dried nanocomposite platform for inhaled rifampicin was reported, in which the powder formulation was obtained after spray-drying a nanosuspension prepared by antisolvent-precipitation and ultrasonication [60].

While formulations based on all of the above delivery systems can be prepared into a dry powder formulation, some liposomal formulations are also suitable for delivery by nebulization of the suspension without subsequent drying [61, 62]. Similarly, a pressurized packed system of rifampicin liposomal formulation in chlorofluorocarbon aerosol propellants has also been reported [63]. Liposomal formulations in pulmonary delivery are desired for targeted delivery of drugs to specific cells such as the alveolar macrophages, to formulate poorly soluble drugs, to control drug release after administration, and to improve drug bioavailability in the lung tissue [61, 64, 65]. Since TB treatment usually requires a high dose of the antibiotic, liposomal systems may not be suitable for formulating anti-TB drugs because of challenges in loading a high amount of drug into the liposomes. On the other hand, liposomes can be utilized for targeted delivery of rifampicin to the alveolar macrophages in the lung. Solid lipid nanoparticles and polymeric nanoparticles are other delivery systems both suitable for pulmonary drug delivery to achieve controlled and targeted delivery of anti-TB drugs [66]. However, both these systems are unsuitable when high-dose delivery of a drug to the lungs is desired. Therefore, among the delivery systems, the microparticle system is most suitable to achieve high-dose delivery of drugs via inhalation since it can be prepared with minimal or no excipients using common techniques like spray drying. Spray drying allows the engineering of particles by manipulation of its process variables, such as the feed characteristics and the operating conditions to obtain small inhalable drug particles (1–5 µm) [67, 68]. Therefore, it is a method of choice for producing both carrier-free dry powder formulations as well as powder formulations composed of engineered particles such as nano-in-micro formulations and porous particles.

### Animal studies of inhaled rifampicin

Studies in animals have been conducted to evaluate the alveolar macrophage uptake, safety and tolerability, toxicity, systemic exposure, pharmacokinetics and bio-distribution, and assessment of antibacterial efficacy after inhaled delivery of rifampicin (Table 3). In vivo studies have mostly used small animals such as mice, rats, and guinea pigs. The dose of delivered drug varies from one animal study to another, but it has consistently been found that inhaled delivery of anti-TB drugs was superior to the oral or intravenous route in achieving higher uptake by alveolar macrophages, higher systemic drug concentrations, and better antibacterial efficacy with minimal or no toxicity to the local lung tissues (Table 3).

**Table 3:** Summary of animal studies for pulmonary delivery of rifampicin either alone or in combination with other drugs

**Anti-TB drug candidate and formulation type**	**Animal species**	**Pulmonary drug delivery technique**	**Dose delivered**	**Purpose of in vivo study**	**Key findings**	**Reference**
Rifampicin microparticles (amorphous and crystalline)	*Sprague Dawley* rats	Intra-tracheal insufflation	25 and 50 mg/kg	Lung and liver safety, tissue distribution, and pharmacokinetics of high-dose inhaled rifampicin	Repeated dose intra-tracheal rifampicin was safe to rat lungs and liver. Intra-tracheal rifampicin resulted in significantly higher bioavailability of rifampicin compared to oral rifampicin at the same dose	[47, 74]
Rifampicin microparticles	Wistar rats	Instillation of suspension via cannulated trachea	1 mL dispersion of rifampicin microparticles in PBS (equivalent to 5 mg/kg rifampicin)	Comparison of rifampicin uptake by alveolar macrophages post oral and pulmonary administration of rifampicin microparticles, and evaluation of hepatotoxicity and phagocytosis activity	Intra-tracheal administration of rifampicin microparticles showed no hepatotoxicity and led to significantly higher rifampicin uptake by alveolar macrophages	[25]
Rifampicin dry powder inhaler (DPI) formulation	Male Wistar rats	Animals allowed to inhale formulation through their sealed mouth	313.56 mg/kg of formulation	Evaluation of toxicity and pulmonary pharmacokinetics	DPI formulation showed negligible toxicity and resulted in higher drug concentration in the lungs, compared to the marketed formulation	[54]
Spray dried mannitol microspheres incorporating rifampicin/PLGA nanoparticles	Male Sprague Dawley rats, 8 weeks old	Administration into the cannulated trachea using a veterinary dry powder insufflator	150 μg/kg rifampicin	Alveolar macrophage uptake of rifampicin in lungs of rats	Nano-sized particles showed higher retention in the lungs. Alveolar macrophage uptake of rifampicin from nano-sized particles was higher compared to micron-sized particles	[114]
Rifampicin-loaded aerosolized liposomes	Male Wistar rats	Animals exposed to aerosolized liposomal formulations and drug only solution, in a nose-only exposure chamber	Average amount of rifampicin delivered per actuation was 45–55 μg and 100 μg for liposomal aerosols and free drug aerosol, respectively	Alveolar macrophage deposition and tissue distribution study	Ligand-anchored liposomal aerosols achieved and maintained high drug concentration in the lung with a high population of alveolar macrophages for a prolonged period	[63]
Nanoparticle based dry powder formulation of rifampicin	Male Wistar rats	Animals allowed to inhale formulation through sealed mouth	313.56 mg/kg of formulations	Pulmonary pharmacokinetic study to investigate the extended local action	Intra-tracheal administration of nanoparticle formulation led to higher *C*_max_ and *t*_1/2_ of rifampicin compared to conventional DPI and marketed formulation, suggesting sustained release profile of nanoparticle formulation	[148]
Rifampicin and isoniazid loaded alginate particles	Female BALB/c mice, 6–8 weeks old	Nebulized delivery of radiolabelled blank carrier system. Intra-tracheal insufflation using an insufflator	Drug dose not mentioned	Lung deposition, pharmacokinetic and bio-distribution studies	Administration of formulated particles led to higher drug levels in the lung and the blood compared to plain drugs	[71]
Rifampicin and rifabutin loaded chitosan microparticles	Female Sprague Dawley rats	Intra-tracheal instillation via incised trachea using a tuberculin syringe attached to 27-gauge needle	200 μL of suspension of microparticles in PBS (equivalent to 1 mg/mL drug)	Acute toxicity study	No significant toxicity to the lungs after intra-tracheal administration of drug-loaded microparticles was observed	[70]
Rifampicin, isoniazid and pyrazinamide encapsulated in PLGA nanoparticles	Dunkin Hartley guinea pigs	A compressor–nebulizer system was used to deliver nebulized formulation	Each animal was exposed to nebulized drugs suspended in 4 mL of 0.9% sodium chloride via a suitable face mask	Evaluation of pharmacokinetics and chemotherapeutic potential against Mtb	A single nebulization to guinea pigs resulted in enhanced bioavailability and sustained drug levels in the plasma and lungs for 6–8 days and 11 days, respectively. There was no bacteria observed in the lung of animals after five doses of nebulized drugs	[72]

*PBS* phosphate buffer saline, *IV* intravenous, *BAL* Bronchoalveolar lavage

Evaluation of the safety and toxicity of high-dose inhaled rifampicin is necessary because a higher dose may result in an increased risk of toxicity to the lung due to potential drug accumulation in the lungs [69]. Rifampicin has proven its potential for inhaled anti-TB therapy, with its safety demonstrated after the pulmonary administration of different types of formulations in laboratory animals [25, 54, 70]. Results from small animal studies have reported that high rifampicin concentrations can be achieved in the lungs (65% dose recovered from the lungs 1 h after administration) as well as in the systemic circulation (AUC_0-24_ of 131 ± 5 µg/mL/h) when delivered via the pulmonary route, further supporting the potential of inhaled rifampicin in TB treatment [71]. Up to 30 and 65% aerosolized rifampicin dose was recovered within 30 min in the serum and the lung, respectively, in a rat study in which various formulations were administered by intra-tracheal delivery [63]. The lack of studies in large animals is due to the challenges associated with experimental complexities, cost, and biosafety requirements. Moreover, most of the anti-TB drugs are well-known compounds rather than new drugs and have well-documented safety and efficacy profiles in humans, albeit via the oral route.

Several studies on inhaled rifampicin have investigated its pulmonary pharmacokinetics, tissue distribution, or toxicity after delivery to the lungs of animals (Table 3). The pulmonary pharmacokinetics of rifampicin have been reported for various formulations to evaluate the efficacy of delivering systemic drug concentrations via the pulmonary route. Comparisons between inhaled and oral rifampicin pharmacokinetics in animals suggest that inhaled rifampicin can achieve similar systemic drug concentrations, resulting in similar bioavailability (AUC_0-∞_ 19.8 ± 7.5 ug·h/mL from inhaled rifampicin compared to AUC_0-∞_ 27.8 ± 10.6 ug·h/mL from oral rifampicin) to that achieved by orally administered rifampicin but at half of the oral dose [48].

Pandey et al. and Suarez et al. reported better therapeutic effects and higher bioavailability from inhaled rifampicin compared to oral rifampicin, suggesting the superiority of inhaled therapy to oral therapy in TB treatment [27, 72]. In the study by Pandey et al. the AUC_0-∞_ of rifampicin from the nebulized formulation was 107 ± 8 mg·h/L, about 12.7 times higher than that from oral rifampicin [72]. In the same study, the nebulized rifampicin required only one administration every 10 days for similar efficacy (Log_10_cfu < 1) in the lung compared to the daily administration required for oral rifampicin. In the study by Suarez et al. nebulized rifampicin microspheres were found to significantly reduce the viable microorganism count (log cfu/mL of 3.8 ± 0.4) in the lung compared to that from the nebulized unformulated rifampicin (log cfu/mL of 4.8 ± 0.1), 4–5 weeks post-infection [27]. However, both studies utilized rifampicin formulations based on poly lactic-co-glycolic acid (PLGA), which were designed for controlled release of the drug and targeted delivery or retention within the lungs. These formulations are not able to deliver high doses of inhaled rifampicin. Moreover, the safety issues specific to formulation excipients or carriers restrict the progress of a formulation to a clinical study. Although a high dose of rifampicin is a requirement for the efficient treatment of TB, studies focusing on a high dose of rifampicin are rare in the literature. Toxicity of inhaled rifampicin to rat lungs was reported previously in two different studies [25, 70]. However, only a low-doses of rifampicin (200 µg and 5 mg/kg) were evaluated in both cases, which have little relevance to clinical conditions because the safety and toxicity evaluation of high-dose inhaled rifampicin in animals is required to determine a safe high-dose for human inhalation during TB treatment. Administration of 5 mg/kg rifampicin via the intra-tracheal route in rats was found to be nontoxic to the liver, as shown by similar serum glutamic-oxalacetic transaminase (SGOT) and serum glutamic-pyruvic transaminase (SGPT) levels before (SGOT: 10.5 ± 0.2 unit/L; SGPT: 62.4 ± 0.9 unit/L) and 240 min after the administration (SGOT: 11.4 ± 0.2 unit/L; SGPT: 72.5 ± 0.8 unit/L), contrary to the significant increase in the enzyme levels observed after the oral administration of rifampicin [25]. Administration of chitosan microparticles equivalent to 200 µg rifampicin by intratracheal instillation resulted only in mild changes in the lung histopathology compared to the severe peribronchiolar infiltration of the inflammatory cells and septal thickening resulted by administration of free unformulated rifampicin at the same dose and route of administration [70]. Another study reported the use of a high-dose (314 mg/kg) rifampicin in rats, in which a passive lung dosing method was adopted for single-dose administration, and the actual amount of drug deposited in the rat lungs was not reported [54]. Nevertheless, the study reported only mild changes in the lung histopathology, suggesting the absence of toxicity after the administration of rifampicin as an inhaled dry powder in rats. In bioavailability studies [48, 73], low-dose rifampicin (5–20 mg/kg) was administered via the inhaled route to rats and guinea pigs, which demonstrated up to two-fold higher bioavailability of rifampicin compared to that from the oral route. In the guinea pig study, administration of 20 mg/kg rifampicin as porous particles via the inhaled route led to a relative bioavailability of 0.87 ± 0.33 compared to the intravenous rifampicin (10 mg/kg), while administration of the same via the oral route at 40 mg/kg dose achieved a relative bioavailability of only 0.61 ± 0.23 [48]. In the rat study, intratracheal administration of 5 mg/kg rifampicin powder formulation was reported to achieve AUC_0-∞_ of 211 μg·h/mL, while the reported AUC_0-∞_ achieved by oral administration of the free drug was 12.0 μg·h/mL. Despite the studies discussed above, the pharmacokinetics of rifampicin after inhaled administration of higher doses (> 20 mg/kg) remained unknown.

These observations suggested a gap in the literature regarding the formulation and animal studies related to inhaled high-dose rifampicin. To address these gaps, a recent study was conducted in laboratory rats to evaluate the safety and pharmacokinetics of high-dose inhaled rifampicin in which the animal doses were determined based on allometric scaling from the estimated human inhalation doses. It was observed that repeated intra-tracheal administration of high-dose rifampicin (up to 50 mg/kg) powder formulations were safe to rat lungs and liver, and intra-tracheal rifampicin decreased the drug burden on the liver compared to oral rifampicin, as suggested by no rise in the serum alanine transaminase (ALT) activity in repeated dose 25 mg/kg and single dose 50 mg/kg intratracheal administration groups compared to the nontreated control group [74]. Similarly, rifampicin powder formulations delivered via the inhaled route resulted in significantly higher systemic bioavailabilities of rifampicin (193.1 ± 37.9 and 126.3 ± 20.3 μg·h/mL) compared to that from oral rifampicin (87.4 ± 64.7 and 71.5 ± 11.0 μg·h/mL) at the same dose after a single administration of 50 mg/kg rifampicin for each formulation, respectively [47].

The following sections will discuss the key considerations for the formulation requirement and preclinical development of inhaled rifampicin for TB treatment. The research gap in the preclinical development of inhalable formulations for TB, along with their challenges, will be discussed.

## Considerations for designing preclinical studies on inhaled rifampicin

### Selection of animal models for preclinical assessment

The selection of appropriate animal species in the preclinical evaluation of inhaled formulations is affected by a number of key factors, such as the anatomical and physiological differences between the animal respiratory system to that of humans, the pathological differences in TB infection between humans and animals, ethical considerations, and financial and zootechnical issues [75]. Moreover, an important difference to animal studies involving drug administration through other routes of administration is that the formulations for pulmonary delivery need to be aerosolized before being administered into the animal body [76]. Therefore, the selection of animal models in pulmonary testing is often driven by the exposure technology available for drug delivery. The animal models used to evaluate formulations for respiratory delivery can mainly be categorized into two groups: small rodents, including mice, rats, guinea pigs, and large mammals, which include rabbits, dogs, sheep, and monkeys [77].

Small animals such as rodents are relatively easier to handle, require small quantities of test material for pulmonary dosing, and more numbers are available for a lower cost and in small-scale facilities. Rodents are more suitable for lung deposition and safety studies of inhaled formulations and have been used for evaluating inhaled pharmacokinetics of drugs. However, due to anatomical differences in their respiratory system compared to that of humans, the results obtained from a rodent study face challenges of extrapolation to human results [78]. Mice, rats, and guinea pigs are all obligatory nose breathers [78], and thus, it is necessary to administer drug formulations directly to their lungs by intra-tracheal administration to assess drug products that are intended to be orally inhaled by humans. The intra-tracheal delivery technique, however, has a limitation in the testing of anti-TB drug formulations or TB disease models due to the requirement of multiple administration of the antibiotic that may last for several weeks and increases the risk of tracheal irritation as well as effects on animal welfare resulting from multiple anesthesia [79]. Long-term testing of anti-TB inhaled formulations in small animals is not feasible, and they are best suited for lung deposition and safety studies and a short-term evaluation of inhaled pharmacokinetics or efficacy after intra-tracheal delivery of anti-TB drugs. For pharmacokinetic studies, repeated blood sampling is necessary, which requires blood vessel cannulation to be performed in animals. The maximum blood volume that can be collected per sample in blood vessel cannulated animals is 0.02 mL, 0.2 mL, and 0.5 mL for mice, rats, and guinea pigs, respectively (Table 4) [80–82]. Considering the volume of samples that can be collected, the use of rats and guinea pigs is more feasible for pharmacokinetic studies in which repeated blood sampling is usually required.

**Table 4:** Total blood volumes and safe blood sampling volumes for laboratory animals

**Animal**	**Reference weight (kg)**	**Blood volume (mL/kg)**	**Total blood volume in normal adult (mL)**	**Safe volume for single bleed (mL)**	**Safe volume from cannulated blood vessel (mL)**
Mouse	0.018–0.040	58.5	Male 1.5–2.4 Female 1.0–2.4	0.1–0.2	0.01–0.02
Rat	0.25–0.5	54–70	Male 29–33 Female 16–19	Male 2.9–3.3 Female 1.6–1.9	0.1–0.2
Guinea Pig	0.7–1.2	69–75	Male 59–84 Female 48–63	Male 6–8 Female 5–6	0.1–0.5
Rabbit	1.0–6.0	57–65	58.5–585	5–50	-
Beagle Dog	-	70–110	900–1170	90–110	-
Sheep	-	58–64	4060–4480	400–450	-
Monkey	-	55–80	Male 420–770 Female 280–630	Male 42–77 Female 28–63	-

References: [81, 82]

Large animals for pulmonary drug delivery testing include rabbits, dogs, sheep, and monkeys, which are used for complex or long-term studies when the establishment of preclinical data is necessary with large doses and lengthy regimens, and analysis is performed on a large volume of biological samples [83]. They tend to have similar respiratory anatomy to humans compared to the smaller animals, although the data obtained from the large animal species still cannot be completely extrapolated to humans due to the influence of laboratory procedures and environments on experimental results, the lack of congruence between human diseases and animal models of diseases, and the interspecies differences in physiology and genetics [84]. Testing of new inhalable formulations and devices in larger species may be required to fulfill regulatory needs when the safety or toxicity of such new entities need to be investigated in at least two different species, one of them being a non-rodent [85]. Due to the technical complexity and requirement of dedicated laboratory facilities, conducting preclinical testing in large animal species is difficult, and as a result, studies reported on in vivo testing of inhaled anti-TB drug formulations in larger species are rare. Nevertheless, preclinical testing of inhaled formulations of drugs of different classes has been conducted in mammals, and the use of rabbits [86, 87], beagle dogs [88–90], and baboons [91] have also been reported.

For preclinical testing of inhaled rifampicin formulations, small animal models are more suitable for lung deposition and safety studies. Since rifampicin has been in clinical use as an anti-TB treatment for several decades and has well-documented systemic safety for its current oral delivery, respiratory tract safety is the primary concern for inhaled delivery. The animal studies are therefore aimed at studying the safety and toxicity to local lung tissues after inhaled delivery. Assessment of the safety of inhaled rifampicin formulations to lung tissues can be conducted in small animals such as mice, rats, and guinea pigs, which allows drug distribution, alveolar macrophage uptake, and pharmacokinetic studies to be conducted.

### Anatomical and physiological differences between human and animal respiratory system

The respiratory differences between humans and laboratory animals in terms of both anatomical layout as well as their physiological parameters have been well discussed in the literature [75, 78, 83, 92–94]. The key differences in the respiratory anatomy and physiology of mammalian species used for preclinical testing in pulmonary drug delivery in comparison to humans are summarized in Table 5.

**Table 5:** Animal species used for preclinical testing of inhaled drugs and their respiratory anatomical and physiological features in comparison to humans

**Animal**	**Human**	**Mouse**	**Rat**	**Guinea pig**	**Rabbit**	**Beagle dog**	**Rhesus monkey**
Body weight (kg)	70	0.02–0.04	0.25–0.35	0.4–1.0	2.5–4.5	10–15	38
Breathing pattern	Nose and mouth breather	Obligate nose breather	Obligate nose breather	Obligate nose breather	Obligate nose breather	Nose and mouth breather	Nose and mouth breather
Airway branching	Relatively symmetric, dichotomous	Monopodial	Strongly monopodial	Monopodial	Strongly monopodial	Strongly monopodial	Monopodial, dichotomous
Number of lung lobes (right and left)	5 (3 and 2)	5 (4 and 1)	5 (4 and 1)	7 (4 and 3)	6 (4 and 2)	6 (4 and 2)	6 (4 and 2)
Lung weight (g)	1000	0.12	1.5	3.2	18	100	46–56*
Trachea length/diameter (cm)	12.0/2.0	0.7/0.12	2.3/0.26	5.7/0.4	6.0/0.5	17.0/1.6	3.0/0.3
Lung volume (mL)	4341	0.74	8.6	13	79.2	736	204
Alveolar surface area (m^2^)	143	0.07	0.4	-	5.8	40.7	-
Lining fluid volume (mL)	2–4	0.005–0.015	0.045–0.055	-	1.22	16.7	-
Number of alveoli (× 10^6^)	950	18	43	69	135	1040	81.8
Respiratory rate (min^−1^)	12	163	85	90	46	18	40
Tidal volume (mL)	400–616	0.15	1.5	1.8	21	11–17	21

References: [78, 83, 92, 93]

^*^For *Macaca arctoides *[172]

The mammalian respiratory tract is known to vary from species to species, with obvious differences in the nasal anatomy and significant differences in the tracheobronchial tree and the lower respiratory system, which affect aerosol deposition, clearance, as well as absorption after inhalation of drug particles [95]. The breathing pattern is also different between species, with rodents (mice, rats, and guinea pigs) and rabbits able to breathe only through their nose, while humans, monkeys, and dogs are capable of breathing through both nose and mouth [93]. This anatomical difference is more likely to be relevant when aerosol delivery to animals involves whole-body exposure or nose-only exposure in which the aerosolized particles need to enter the animal lung via the nasal opening. Anatomical differences further down the respiratory tract include differences in airway branching, lung weight and symmetry, tracheal and tracheobronchial length and diameters, lung volume, the number and size of alveoli, and number of alveolar macrophages [78]. Similarly, the physiological differences between species include the differences in their respiratory rates, tidal volumes, and ventilation rates [93].

Consideration of the differences in respiratory anatomy and physiology between the selected animal model and humans is important for inhaled drug and formulations. This allows allometric conversion of the effect observed and responses recorded in animals into that for humans.

### Methods for pulmonary drug administration to animals

The complexity of the respiratory system makes it challenging to deliver drug aerosols efficiently to the desired site in the respiratory tract of laboratory animals. In most cases, anesthetizing or restraining the animals is inevitable, which may alter the normal respiratory phenomena or induce stress in animals. Nevertheless, the development of state-of-the-art tools and techniques has allowed the precise delivery of drug aerosols to laboratory animals with minimized risk while maintaining animal welfare to the best possible standard.

The pulmonary drug delivery technique to animals depends on the nature of the study and its goals. The delivery techniques are categorized into either direct or passive techniques depending on whether aerosol is administered to the animal or the animal inhales the aerosol by breathing, respectively [78, 92]. Direct delivery techniques are suitable when a predetermined amount of drug is to be delivered to the lungs, while passive techniques are preferable to study the aerodynamics of drug particles in the respiratory tract together with the drug effects after deposition.

#### Direct pulmonary administration

**Fig. 1:**
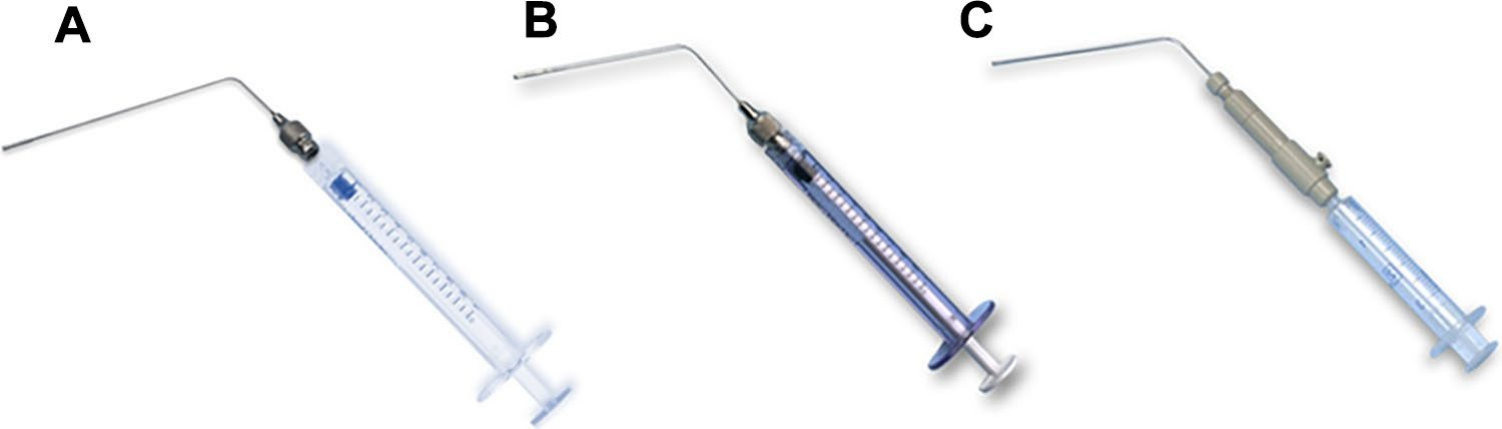
Devices for direct aerosol delivery to small animals. **A** Device for intra-tracheal liquid instillation; **B** Microsprayer^®^ Aerosolizer for liquid aerosol delivery; **C** dry powder insufflator. B and C were manufactured by Penn-Century, Inc

**Fig. 2:**
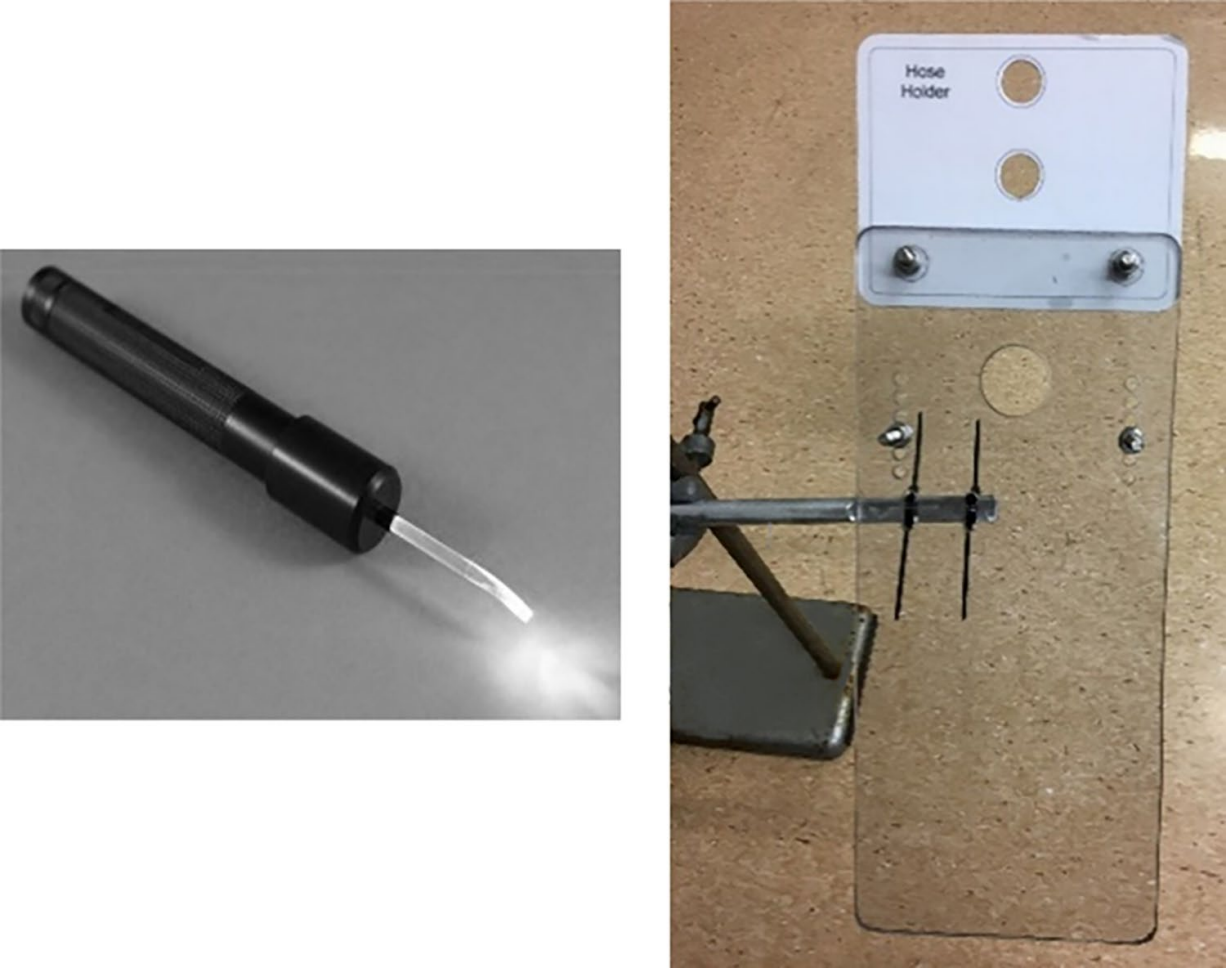
A small animal laryngoscope (left) and a prototype rat intubation platform (right)

Direct administration is often carried out under anesthesia. This technique allows the delivery of desired doses of drugs with minimum loss while being accurately measured. The common methods of direct aerosol administration in animals are liquid instillation, liquid spray instillation, and dry powder insufflation to deliver a liquid bolus, liquid spray, and powder aerosol, respectively. The delivery devices commonly employed for direct aerosol delivery to small animals are known as sprayers or insufflators (Fig. 1). Recently, a Venturi-effect device for direct delivery of aerosolized anti-TB inhalable powders for uniform lung distribution was reported by Hirota et al. in which the Venturi-effect administration showed three times higher drug administration compared to a conventional insufflator [96]. The animals need to be anesthetized and laid in a supine position on a suitable platform to visualize the vocal cords and the trachea with the help of a laryngoscope [93]. The tip of the aerosol device is then inserted into the trachea up to the carina, and the device is actuated to release the aerosol. A small animal laryngoscope (manufactured by Harvard Apparatus, Holliston, MA, USA) and a prototype rat intubation platform, designed in-house, are shown in Fig. 2. Direct administration of drug formulations to the airways of small animals can also be undertaken in anesthetized, tracheotomized, and mechanically ventilated animals using a purpose-built jet nebulizer or a dry powder delivery apparatus [97]. For direct aerosol delivery to larger animals, anesthetic and surgical procedures are often required [92]. Delivery of liquid drug formulations and powder aerosols to large animals is performed with dedicated sprayers and dry powder inhaler devices, respectively, via oro-tracheal intubation [75].

The use of direct delivery methods in animals is invasive and requires the use of anesthesia and surgical procedures. This can cause irritation to the animal respiratory tract and induces stress in animals. Therefore, repeated or long-term pulmonary administration of drug formulations by this method is challenging.

#### Passive pulmonary administration

**Fig. 3:**
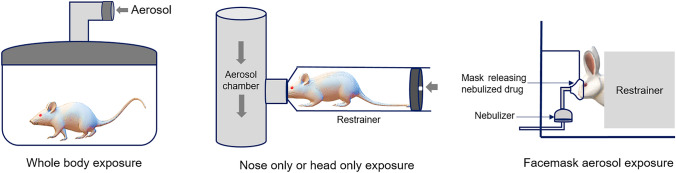
Exposure systems for passive pulmonary drug administration to laboratory animals

Passive administration of aerosols to animals is undertaken by exposing them to an aerosol chamber where both conscious and unconscious animals can breathe in the aerosolized drugs following either a whole-body, head-only, nose-only, or facemask exposure (Fig. 3). The limitations of this method include variation in dose delivered to the respiratory tract, a requirement of large doses to compensate for drug loss, the possibility of drug absorption by cutaneous or oral routes, the requirement of an aerosol generator, and size limitations of exposure chamber for larger animals [78, 92].

After whole body exposure, the dose delivered to the animal can be calculated using Eq. 1, where *D* refers to the delivered dose (mg/kg), *C* is the drug concentration in air (mg/L), RMV is the respiratory minute volume of the animal (L/min), *T* is the duration of exposure (min), RF is the respirable fraction of the formulation, and BW is the weight of the animal (kg) [83, 98].

1 $$D=\frac{C\; x\;\mathrm{RMV }x\; T\; x\;\mathrm{RF}}{\mathrm{BW}}$$

Passive pulmonary administration of drug formulations to large mammals can be carried out via face mask inhalation, but this technique also has limitations due to the requirement of animal training and difficulties in predicting lung deposition and thus can be used only for lung tolerance or regulatory toxicity assessment for long-term studies [75].

Between the two different delivery techniques, direct aerosol delivery is preferable in testing inhaled powder or liquid formulations in animals since it allows precise delivery of higher doses to animal lungs, which is a key requirement for rifampicin. The passive delivery technique is not suitable for studies on high-dose rifampicin formulations since the animals are not able to inhale large doses of aerosolized drug particles on their own [93]. Toxicity evaluation of low rifampicin doses, on the other hand, will not be relevant for extrapolation to human responses, which is expected to involve the use of high inhaled rifampicin doses for TB treatment.

### Assessment of the in vivo properties of the aerosol

**Fig. 4:**
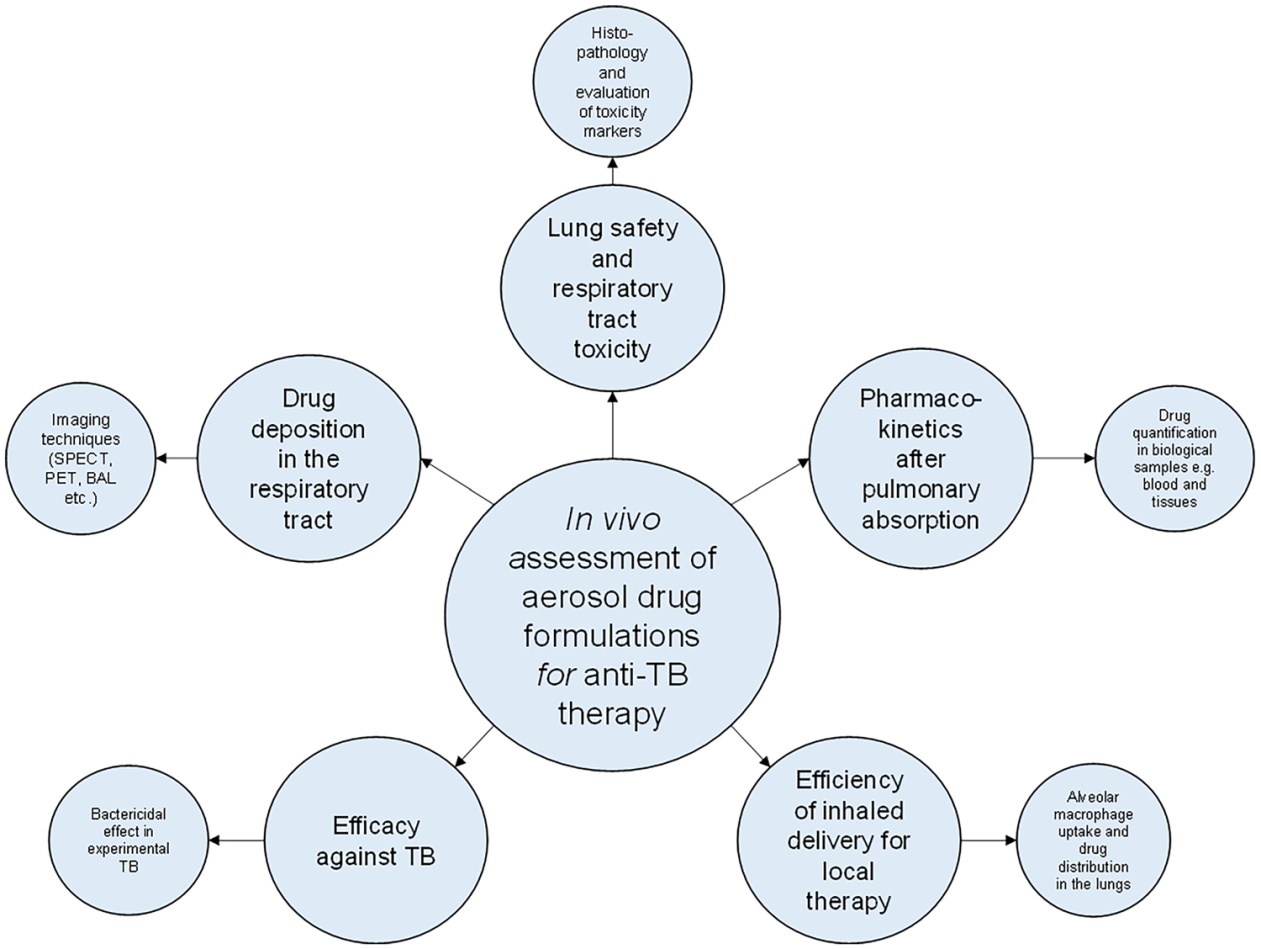
Assessment of in vivo properties of aerosol drug formulations developed for anti-TB therapy

Assessment of in vivo properties of drug formulations delivered via the pulmonary route mainly includes an assessment of the particle deposition, safety, and toxicity to the lung and other organs, absorption and disposition after pulmonary delivery, or the efficacy after inhaled therapy, often known as the endpoints of a preclinical study in animals (Fig. 4).

The drug deposited after pulmonary delivery to laboratory animals can be determined either by imaging techniques such as gamma scintigraphy, single-photon emission computed tomography (SPECT), and positron emission tomography (PET) or by bronchoalveolar lavage (BAL) procedure [83]. For imaging techniques, the drug particles often need to be conjugated with radioligands to be imaged. The BAL procedure is an invasive method in which normal saline is injected into the lung, followed by its aspiration to collect deposited drug along with cells, soluble proteins, lipids, and other chemical constituents from the epithelial region [99]. The procedures to measure drug deposition after pulmonary delivery should be performed early enough to assure accurate measurement of the deposited drug before subsequent drug absorption, clearance, or metabolism [83].

The endpoint of safety and toxicity evaluation of inhaled drug formulation in animals is histopathology, which includes histopathologic examination of lung tissues and a range of organs for evaluation of local and systemic toxicities, respectively [100]. Similarly, markers of pulmonary inflammation such as lactate dehydrogenase activity for tissue injury, protein levels for permeability alteration and inflammatory cytokines such as TNF-*α* can be examined from BAL fluid to assess the safety of the inhaled formulations [93].

The measurement of drug absorption and disposition after pulmonary delivery in animals is possible by measurement of drug concentration in the plasma as well as the tissues. The concentration in the tissues provides a tissue distribution profile of the drug after pulmonary delivery. Absorption and elimination profiles of the drug in plasma after inhaled delivery can be studied by pharmacokinetic evaluation, which is essential for drugs intended for systemic action, as well as in bioequivalence studies for comparison between two inhaled formulations. The pharmacokinetic evaluation permits an understanding of the fate of the drug from its inhaled formulation in terms of its area under the plasma concentration–time curve (AUC), the maximum plasma concentration (*C*_max_), the time to *C*_max_ (*T*_max_), absorption and elimination rates, and bioavailability of the inhaled drug. Estimation of pharmacokinetics after pulmonary administration of a drug formulation is often carried out using compartmental, non-compartmental, and physiologically based pharmacokinetic (PBPK) models [83]. A compartmental model assumes a system composed of a group of arbitrarily sized compartments, where one of them is assigned as the central compartment to which absorption of the drug occurs and is also the site from which the drug gets excreted. The non-compartmental approach is simpler and usually followed when data are limited to the drug concentration versus time in a single compartment. Similarly, the PBPK model consists of multiple compartments in which each compartment is a defined space within the body and considers the anatomy, physiology, direction of blood flow, rates of drug extraction, or metabolism relevant to the actual physiological conditions for each compartment.

Among the key assessments of in vivo properties of inhaled drugs in animals, the efficacy assessment is the most important. While endpoints for assessment of efficacy are different based on the type of the disease, the efficacy endpoint for TB is either the reduction in bacterial burden or complete eradication of the bacteria. Comparison of efficacy between inhaled and orally administered anti-TB drugs in an animal model is rare in the literature, possibly due to complexity in the experimental requirements as well as the pathogenesis of the disease itself. TB pathogenesis is known to be different in humans and animals, with distinct pathological features such as primary and secondary granuloma formation, cavity formation with necrosis, and caseation being prominent in humans while absent in some laboratory animals [76]. This makes the extrapolation of animal efficacy results in humans more difficult. Nevertheless, assessment of the efficacy of inhaled drugs against TB in animal models is necessary to strengthen the hypothesis that pulmonary delivery of anti-TB drugs is potentially effective for TB treatment. This can be best achieved by selecting the animal model with the highest similarity to humans in terms of TB pathogenesis. For this purpose, guinea pigs have been widely used as the model of choice because their TB infection features human-like pathological changes such as granulomas with central necrosis within the immune cells and a fibrotic capsule [101].

### Selection of the right inhaled formulation for in vivo testing

The formulations used for testing in laboratory animals via the pulmonary route include dry powder and liquid formulations for nebulization intended for human use. Animal experiments do not take into account the effect of inhaler devices that are used in clinical practice. This might have an effect on the assessment of drug deposition patterns in the respiratory tract of animals. However, this does not hinder the studies intended for the evaluation of safety, pharmacokinetics, drug distribution, or efficacy. The formulations are therefore designed with the intended features for clinical use in humans regardless of the technical difficulties in animal studies. Due to this reason, drugs can sometimes be administered in liquid form to animals for safety evaluation, even though the intended formulation for human use may be a dry powder inhaler [25, 70].

### Dose calculation for in vivo testing

In the case of passive pulmonary administration of aerosolized formulations to animals, the dose delivered to the airways mainly depends on the factors associated with Eq. 1. Among all the variables, the respiratory minute volume (RMV) is the animal-dependent variable that affects the dose of the aerosol deposited. RMV is calculated using an allometric equation based on body weight (BW in kg) (Eq. 2) [98].

2 $$\mathrm{RMV }(\mathrm{L}/\mathrm{min})=0.608 \times {\mathrm{BW}}^{0.852}$$

For studies dealing with direct pulmonary administration, the selection of inhaled dose varies based on the study design and the experimental feasibility. As a result, there is a lack of consistency in the dose of a specific drug delivered to the animal lungs in the reported studies (Table 3). While there are no criteria developed for the calculation of inhaled doses to be delivered in animal studies, the current approach is based on individual experimental design and feasibility. The maximum dose and duration in toxicological studies via the pulmonary route may be set depending on the anatomy and physiology of the animal respiratory system. However, one approach to determine the prospective animal inhaled doses is to consider the allometric scaling for dose conversion. Allometric scaling is based on the normalization of dose-to-body surface area and is often used for dose extrapolation in determining maximum starting human doses for clinical studies. This has been established as a guidance by the USFDA for dose conversion between humans and animals to calculate equivalent human or animal doses [102]. Based on the allometric scaling, the human equivalent dose (HED) can be calculated as shown in Eq. 3.

3 $$\mathrm{HED}\left(\frac{\mathrm{mg}}{\mathrm{kg}}\right)=\mathrm{Animal\ dose}(\mathrm{mg}/\mathrm{kg}) \times {\left(\frac{\mathrm{weight\ of\ animal\ in\ kg}}{\mathrm{weight\ of\ human\ in\ kg}}\right)}^{(1-0.67)}$$

The exponent for body surface area (0.67) in Eq. 3 accounts for the difference in metabolic rates and body surface area between animals and humans. Usually, the animal dose in Eq. 3 is the no observed adverse effect level (NOAEL) in animals determined from the toxicological study to calculate HED. The maximum starting dose in humans is usually one-tenth of the HED calculated based on NOAEL.

For determining animal inhaled doses in preclinical studies, the animal doses can be determined from Eq. 3 by estimating the expected dose in humans. This approach can be helpful in preclinical safety and toxicity studies of inhaled drugs, in which the safety or toxicity profile of the relevant animal dose needs to be established with respect to the estimated human dose. For pharmacokinetics and efficacy studies of inhaled drugs, using a relevant dose in animals can be helpful in establishing reliable preclinical data for future clinical studies.

### Regulatory considerations on the development of inhaled formulations

The guidelines for manufacturing and quality control of inhalation drug products have been developed by the FDA and the European Medicines Agency (EMA) for metered dose inhalers, dry powder inhalers, as well as solutions and suspensions for nebulization [103–105]. The preclinical studies on the inhaled formulations, however, need to establish evidence of safety and efficacy in animal models through the pulmonary route. The safety assessment for a new drug requires studies in two different species, including rodent and a non-rodent model [85]. Development of inhalable formulations against TB usually includes an approved anti-TB drug, conventionally administered via the oral or parenteral routes. However, when an approved drug is to be administered via a different route other than its previously approved route of administration, it is generally considered a new drug entity by the regulatory bodies. Therefore, preclinical development of inhaled formulations of currently used anti-TB drugs may face the requirement of rigorous safety and toxicity studies in preclinical models, similar to that for new drugs under development.

A decision-making process known as regulatory nonclinical safety evaluations (RNSE) is used by regulators to assess if an inhaled drug product can be safely used in humans based on the available non-clinical data on the drug product [98]. RNSE determines whether the safety margins of the inhaled drug product are acceptable (both the inhaler device and the formulation) for its intended clinical doses and is a complex decision-making process that takes into account the formulation features, the inhaler device characteristics, and the results from inhaled toxicology study in animals.

## The research gaps hindering the transition of inhaled rifampicin from preclinical development to clinical investigation

Studies evaluating inhaled formulations in animals require consideration of several factors, including animal species, drug formulation, inhaled delivery technique, the dose of the drug to be tested, and the parameters of interest together with their endpoints based on the intended clinical use. Only a few studies have reported the testing of a high dose of anti-TB drugs in animals, which is an important consideration in designing, developing, and testing inhaled formulations for TB [106]. Similarly, the inhaled doses of rifampicin reported in the animal studies are not determined based on the intended clinical dose for humans, as no allometric conversion of human and animal doses was reported. On the other hand, some of the studies have not reported the technique of inhaled drug administration to animals [107–109] or the dose of inhaled drug [110]. Thus, a research gap between the preclinical testing and the clinical evaluation exists for inhaled rifampicin since the majority of the formulation and animal studies reported in the literature have not considered the requirement of the high dose and the importance of animal dose selection. It is important to plan and conduct in vivo studies on inhaled rifampicin in such a way that the findings in animals can be utilized as the basis for further clinical evaluation.

Apart from the case of inhaled rifampicin, the gap between the large number of studies related to formulation development and the much smaller number of clinical studies involving inhaled anti-TB drugs has left several questions unanswered for inhaled anti-TB therapy. One reason for the few clinical studies on inhaled anti-TB formulations might be the lack of enough in vivo preclinical data on the safety, pharmacokinetics, and efficacy of inhaled anti-TB formulations. Nevertheless, a number of antibacterial drugs, such as ciprofloxacin, tobramycin, and colistimethate have been found to be safe and effective in treating respiratory infections in patients when delivered by inhalation [111]. Such evidence from clinical studies provides confidence for further preclinical and clinical evaluations of inhaled anti-TB drugs in the future. Preclinical evaluations of inhaled anti-TB formulations are necessary to answer key questions regarding the safety, pharmacokinetics, and efficacy of anti-TB drugs delivered via the inhalation route. The findings from such studies can inform clinical studies on the expected dose, the expected tissue and blood concentration of the drugs after inhaled administration, and clinical efficacy compared to the current anti-TB regimen.

## Conclusions

Among the anti-TB drugs investigated for their potential inhaled delivery, rifampicin has been extensively investigated as micro-particles, liposomes, solid lipid nanoparticles, polymeric nanoparticles, porous particles, nanoaggregates, and nanocomposites for delivery via the inhalation route. Some of these formulations of rifampicin were also studied in animals to evaluate in vivo properties of the drug after inhaled delivery. However, none of the formulations have progressed to clinical trials.

A lack of well-designed preclinical studies has created a gap between formulation studies and clinical trials. A comparison of inhaled versus oral or intravenously administered drugs needs to be performed in preclinical models at a dose relevant to the intended future human dose. And there must be a careful evaluation of local lung toxicity and pharmacokinetics in comparison to the conventional route of administration for the drug. If this bottleneck in the drug pipeline can be addressed, the promise of inhaled drug formulations for anti-TB therapy may be fulfilled.

## Data Availability

Data sharing is not applicable to this article as no datasets were generated or analyzed during the current study.

## Materials availability

Data sharing is not applicable to this article as no datasets were generated or analyzed during the current study.
